# Hormone Receptor Expression Analyses in Neoplastic and Non-Neoplastic Canine Mammary Tissue by a Bead Based Multiplex Branched DNA Assay: A Gene Expression Study in Fresh Frozen and Formalin-Fixed, Paraffin-Embedded Samples

**DOI:** 10.1371/journal.pone.0163311

**Published:** 2016-09-20

**Authors:** Annika Mohr, Florenza Lüder Ripoli, Susanne Conradine Hammer, Saskia Willenbrock, Marion Hewicker-Trautwein, Zdzisław Kiełbowicz, Hugo Murua Escobar, Ingo Nolte

**Affiliations:** 1 Small Animal Clinic, University of Veterinary Medicine Hannover, Foundation, Hannover, Germany; 2 Division of Medicine Clinic III, Hematology, Oncology and Palliative Medicine, University of Rostock, Rostock, Germany; 3 Institute of Pathology, University of Veterinary Medicine Hannover, Foundation, Hannover, Germany; 4 Department and Clinic of Veterinary Surgery, Faculty of Veterinary Medicine, Wroclaw University of Environmental and Life Sciences, Wrocław, Poland; Bauer Research foundation, UNITED STATES

## Abstract

Immunohistochemistry (IHC) is currently considered the method of choice for steroid hormone receptor status evaluation in human breast cancer and, therefore, it is commonly utilized for assessing canine mammary tumors. In case of low hormone receptor expression, IHC is limited and thus is complemented by molecular analyses. In the present study, a multiplex bDNA assay was evaluated as a method for hormone receptor gene expression detection in canine mammary tissues. Estrogen receptor (ESR1), progesterone receptor (PGR), prolactin receptor (PRLR) and growth hormone receptor (GHR) gene expressions were evaluated in neoplastic and non-neoplastic canine mammary tissues. A set of 119 fresh frozen and 180 formalin-fixed, paraffin-embedded (FFPE) was comparatively analyzed and used for assay evaluation. Furthermore, a possible association between the hormone receptor expression in different histological subtypes of canine malignant mammary tumors and the castration status, breed and invasive growth of the tumor were analyzed. The multiplex bDNA assay proved to be more sensitive for fresh frozen specimens. Hormone receptor expression found was significantly decreased in malignant mammary tumors in comparison to non-neoplastic tissue and benign mammary tumors. Among the histological subtypes the lowest gene expression levels of ESR1, PGR and PRLR were found in solid, anaplastic and ductal carcinomas. In summary, the evaluation showed that the measurement of hormone receptors with the multiplex bDNA assay represents a practicable method for obtaining detailed quantitative information about gene expression in canine mammary tissue for future studies. Still, comparison with IHC or quantitative real-time PCR is needed for further validation of the present method.

## Introduction

Although IHC is considered the method of choice for analyzing hormone receptor status in human breast cancer [1], studies are prone to variability due to variation in antibody clones and assay interpretation [2]. Especially when evaluating hormone receptors in canine mammary tumors (CMT) the percentage of detected hormone receptor-positive tumors varies greatly among the studies because of the different monoclonal antibodies and scoring systems used [3]. As IHC is limited in the case of low receptor expression [4], a more sensitive method is beneficial for a detailed assessment of differences in gene expression. Furthermore, multiplexing approaches are convenient for a rapid diagnostic process and for avoiding tissue consumption [5]. Although quantitative real-time PCR (qRT-PCR) is a common method for gene expression analysis, it is limited by its low multiplex capacity [6]. In addition, a previous study revealed its restriction when analyzing FFPE specimens compared to a branched DNA (bDNA) assay [7]. In the present study, a multiplex bDNA assay was evaluated as a method for hormone receptor gene expression detection in canine mammary tissues. In comparison to qRT-PCR, the bDNA assay amplifies the signal instead of the target gene [8]. Thus, the target RNA is detected directly. Additionally, the bDNA assay does not depend on a pre-amplification of the nucleic acid (which is a major source of gene-specific measurement errors). Moreover, the simple assay format with consequently decreasing pipetting errors is a major advantage of the bDNA assay in comparison to qRT-PCR [9]. The combination of bDNA assay and xMAP^®^ Luminex^®^ magnetic beads of the multiplex bDNA assay enables amplification-free and quantitative determination of up to 100 genes from one sample [6]. Furthermore, its sensitivity also allows small quantities of specimens to be analyzed.

Until now only estrogen receptor (ESR1) and progesterone receptor (PGR) have been routinely assessed as hormone receptors in human breast cancer due to their prognostic and predictive value in human mammary carcinoma therapy [1]. Nevertheless, other hormone receptors such as prolactin receptor (PRLR) or growth hormone receptor (GHR) have been evaluated in human breast cancer [10–11]. As approximately 50% of mammary gland tumors in dogs appear to be malignant [12–13], the detection of novel canine tumor markers with a value for prognosis or targeted therapy are the focus of research [14–19]. Therefore, in the present study, the gene expression of ESR1, PGR, PRLR and GHR were analyzed simultaneously to compare their distribution in canine mammary tissue. Both fresh frozen and FFPE specimens were analyzed to enlarge the sample size, but also to evaluate if FFPE samples are suitable for future gene expression studies and as reliable as fresh frozen samples using the multiplex bDNA assay. Besides, it should be analyzed whether there was an association between hormone receptor gene expression in different histological subtypes of canine malignant mammary tumors and the castration status, the breed, and invasive growth of the tumor.

## Materials and Methods

### Patient’s material

In total 299 mammary tissue samples of 259 dogs were used comprising 119 fresh frozen and 180 FFPE samples. Fresh frozen tissue samples were collected during routine mastectomy between 2003 and 2011 at the Small Animal Clinic, University of Veterinary Medicine Hannover, Foundation, Germany and in 2014 at the Clinic for Small Animals, Institute of Veterinary Medicine, Georg-August-University, Göttingen, Germany. The samples were snap frozen using liquid nitrogen and archived for long-term storage at -80°C until further usage. Representative parts of the fresh frozen tissues were fixed in 10% neutral buffered formalin, embedded in paraffin wax and routinely processed for the histological classification. FFPE samples were retrospectively retrieved from the archives of the Institute of Pathology of the University of Veterinary Medicine Hannover, Foundation. The FFPE specimens were obtained surgically between 1993 and 2000 at the Small Animal Clinic, University of Veterinary Medicine Hannover, Foundation. All herein utilized tissue samples were removed with informed consent of the owner. Therefore, ethical approval was not necessary according to the German regulations.

### Histological classification

Sections of 3–4 μm were stained with hematoxylin and eosin (H-E), classified by a pathologist and subcategorized into the following groups: group 1: non-neoplastic mammary tissue, group 2: mammary hyperplasia/dysplasia, group 3: benign mammary tumors, group 4: malignant mammary tumors. Additionally, the tumors were classified into different histopathological tumor subtypes according to Goldschmidt et al. [20].

### RNA isolation

Prior to RNA isolation, the fresh frozen samples were homogenized using a TissueLyser II (Qiagen GmbH, Hilden, Germany) and 5mm stainless steel beads. The RNA was isolated with the RNeasy Mini Kit (Qiagen GmbH, Hilden, Germany) with an additional step of digestion using the RNase-free DNase Set (Qiagen GmbH, Hilden, Germany) according to the manufacturer’s protocol. Additionally, genomic DNA was digested using RQ1 RNase-free DNAse (Promega GmbH, Mannheim Germany). Total RNA was quantified using the Synergy multi-mode reader and the Gen5™ Reader Control and Data Analysis Software (Biotek, Bad Friedrichshall, Germany) following storage at -80°C until further usage.

From the FFPE samples 20 μm sections were cut with a microtome (pfm Slide 2003, pfm medical AG, Köln, Germany) and stored until further usage in RNAse free Eppendorf Cups (Eppendorf, Hamburg, Germany). Deparaffinization Solution (Qiagen GmbH, Hilden, Germany) was used prior to RNA purification with the AllPrep DNA/RNA FFPE Kit (Qiagen GmbH, Hilden, Germany) following manufacturer’s instructions. Total RNA was quantified using the Synergy multi-mode reader and the Gen5™ Reader Control and Data Analysis Software (Biotek, Bad Friedrichshall, Germany) following storage at -80°C until further usage.

### QuantiGene 2.0 Plex Assay

Gene expression was analyzed using the QuantiGene 2.0 Plex Assay (Affymetrix, Santa Clara, USA). For each gene (ESR1, PGR, PRLR and GHR) and the housekeeping genes, Glyceraldehyde 3-phosphate dehydrogenase (GAPDH), Hypoxanthin-Phosphoribosyl-Transferase 1 (HPRT1) and Beta-actin (ACTB), specific bead-based oligonucleotide probe sets (Accession Numbers listed in Table 1) were custom designed by Affymetrix (Santa Clara, USA). The assay was performed according to the manufacturer’s protocol. Briefly, 20 μL extracted total RNA at 250 ng for each sample of fresh frozen specimens and 200 ng of FFPE specimens was mixed with 80 μL of Working Bead Mix containing Probe Sets (5 μL) with Capture Beads (1 μL), Lysis Mixture (33.3 μL), Blocking Reagent (2 μL) and Nuclease-free Water (38.7 μL) for each well. The reactions were placed in a 96-well Hybridization Plate and incubated at 54°C for 18 h at 600 rpm in a VorTemp shaking incubator (Labnet International, Edison, New Jersey, USA). Afterwards, the 96-well Hybridization Plate was transferred to a Magnetic Separation Plate to fix the magnetic beads at the bottom of the well and washed 3 times with washing buffer to remove unbound material.

**Table 1:** List of genes and their respective Accession Number and Probe Set Region used in the QuantiGene 2.0 Plex Assay.

Accession Number	Symbol	Probe Set Region
**Analyzed genes**		
NM_001286958	ESR1	921–1321
NM_001003123	GHR	1286–1741
NM_001003074	PGR	1850–2359
XM_536502	PRLR	1460–1883
**Housekeeping genes**		
XM_536888	ACTB	577–903
NM_001003142	GAPDH	258–726
NM_001003357	HPRT1	15–553

Three series of hybridizations were performed at 50°C for 1 h at 600rpm with 100 μL of Pre-Amplifier, 100 μL of Amplifier and 100 μL Label Probe Solution, respectively, followed by 3 washes with washing buffer after each incubation. To develop the amplified signal, streptavidin phycoerythrin (SAPE) Working Reagent was added to the wells and incubated at room temperature for 30 minutes at 600 rpm. Then, wells were washed with SAPE Wash Buffer and analyzed by using a Luminex® 100/200™ System (Luminex Corporation, Austin, Texas, USA). The signal (expressed as mean fluorescence intensity, MFI) of each bead is proportional to the amount of target RNA in the sample.

### Data analysis and statistical analysis

The median fluorescence was subtracted by the background and normalized to the average of the housekeeping genes GAPDH, HPRT1 and ACTB. Following manufacturer’s instructions, 15 FFPE samples were below the limit of detection (Background plus 3 standard deviations of the background) and therefore had to be excluded from the analysis. Furthermore, the ESR1 levels of 5 fresh frozen and 10 FFPE samples and the PGR levels of 1 fresh frozen and 2 FFPE samples were below the limit of detection. The PRLR levels of 4 fresh frozen and 10 FFPE samples and the GHR levels of one fresh frozen sample were below the limit of detection. Consequently, these samples were excluded from the analysis.

Statistical analysis was performed using the SAS Enterprise Guide 7.1 (SAS Institute Inc., Cary, North Carolina, USA). The data were tested for normal distribution and found not to be normally distributed. Therefore, statistical significance was tested using the Mann-Whitney-U-Test. Significance was defined at p<0.05.

As the number of the non-neoplastic tissue of FFPE samples (n = 4) and mammary hyperplasia/dysplasia in fresh frozen samples (n = 3) was too small, they were not used for statistical analysis. Therefore, non-neoplastic mammary tissue was chosen as the reference tissue for fresh frozen samples and mammary hyperplasia/dysplasia as the reference tissue for FFPE samples, respectively.

## Results

### Samples

284 samples of 244 dogs were included in the final analysis.

119 fresh frozen samples of 98 dogs were analyzed. 15 patients were represented with more than one sample of mammary tissue. The age of the dogs at the time of the tumor removal ranged from 5 to 15 years (mean: 9.99 years). 83 of the dogs were intact and 15 neutered. 33 different breeds were represented in the fresh frozen samples with crossbreeds (n = 21 [21%] dogs), Terrier (n = 13 [13%] dogs) and Dachshund (n = 12 [12%] dogs) representing the most common breeds.

165 FFPE samples of 146 female dogs were used. Of 15 patients more than one sample was analyzed. The age of the dogs at the time of tumor removal ranged from 1 to 15 years (mean age: 9.7 years). 13 samples were taken from neutered females. 26 different breeds took part in the study. Crossbreeds (n = 35 [24%] dogs), Dachshund (n = 24 [16%] dogs), Terrier (n = 15 [10%] dogs), Cocker Spaniel (n = 13 [9%] dogs), and German Shepherd (n = 11 [7.5%] dogs) were the most common breeds.

### Histopathology

The 119 fresh frozen samples included 15 non-neoplastic mammary tissues, 3 mammary hyperplasia/dysplasia, 33 benign mammary tumors, and 68 malignant mammary tumors.

Of the 165 FFPE samples, 4 were non-neoplastic mammary tissue, 20 mammary hyperplasia/dysplasia, 47 benign mammary tumors, and 94 malignant mammary tumors. As some of the FFPE blocks contained different tumors, the diagnosis with the most severe pathological features was taken for categorizing them into the groups for the analysis.

For further subcategorization into histological subtypes [20], only FFPE blocks with a clear/plain histopathological diagnosis were used (Table 2). As 13 blocks contained more than one histopathological diagnosis they were excluded from the analysis of the histological subtypes.

**Table 2:** Details of histopathological subtype of reference tissue and malignant mammary tumors, invasive growth and castration status of the canine mammary tissue studied. Sample number is shown for fresh frozen and FFPE specimens.

histological subtype	total	Fresh frozen (intact/neutered)	FFPE (intact/neutered)	invasive growth (fresh frozen/FFPE)
non-neoplastic mammary tissue	19	15 (14/1)	4 (1/3)	
lobular hyperplasia	13	3 (3/0)	10 (9/1)	
simple carcinoma	27	13 (11/2)	14 (12/2)	7 (2/5)
solid carcinoma	15	8 (6/2)	7 (5/2)	8 (3/5)
comedocarcinoma	3	1 (1/0)	2 (2/0)	2 (0/2)
anaplastic carcinoma	11	8 (5/3)	3 (2/1)	1 (1/0)
carcinoma arising in a benign tumor	18	10 (10/0)	8 (7/1)	0
carcinoma complex type	24	13 (11/2)	11 (10/1)	2 (1/1)
carcinoma and malignant myoepithelioma	8	1 (1/0)	7 (7/0)	4 (0/4)
carcinoma mixed type	4	2 (2/0)	2 (2/0)	1 (1/0)
ductal carcinoma	20	5 (4/1)	15 (15/0)	9 (1/8)
intraductal papillary carcinoma	21	1 (1/0)	19 (19/0)	7 (0/7)
squamous cell carcinoma	3	2 (1/1)	1 (0/1)	1 (0/1)
adenosquamous carcinoma	2	1 (1/0)	1 (1/0)	0
osteosarcoma	1	1 (1/0)	0	0
chondrosarcoma	1	0	1 (1/0)	0
fibrosarcoma	1	1 (1/0)	0	0
other sarcomas	1	1 (1/0)	0	0

### Gene expression analyses

#### Comparison of gene expression between fresh frozen and FFPE samples

Comparison of ESR1 gene expression regarding the diagnosis or the histological subtypes revealed no significant difference between the fresh frozen and FFPE samples. PGR expression showed significant differences when comparing benign mammary tumors (p = 0.005), simple tubular carcinomas (p = 0.003) and carcinomas arising in a benign tumor (p = 0.024) of fresh frozen and FFPE samples with a higher expression in fresh frozen samples. PRLR expression showed no significant differences between fresh frozen and FFPE samples except for the group of malignant mammary tumors (p = 0.048) with a higher expression in FFPE samples. In benign (p<0.0001) and malignant mammary tumors (p = 0.009) significant differences were observed in the gene expression of GHR with a higher expression in FFPE samples.

#### Hormone receptor gene expression

All hormone receptors showed significantly lower expression levels in malignant mammary tumors compared to benign mammary tumors, mammary hyperplasia/dysplasia of FFPE samples, and non-neoplastic mammary tissue of fresh frozen samples (S1–S4 Figs).

**Fig 1:**
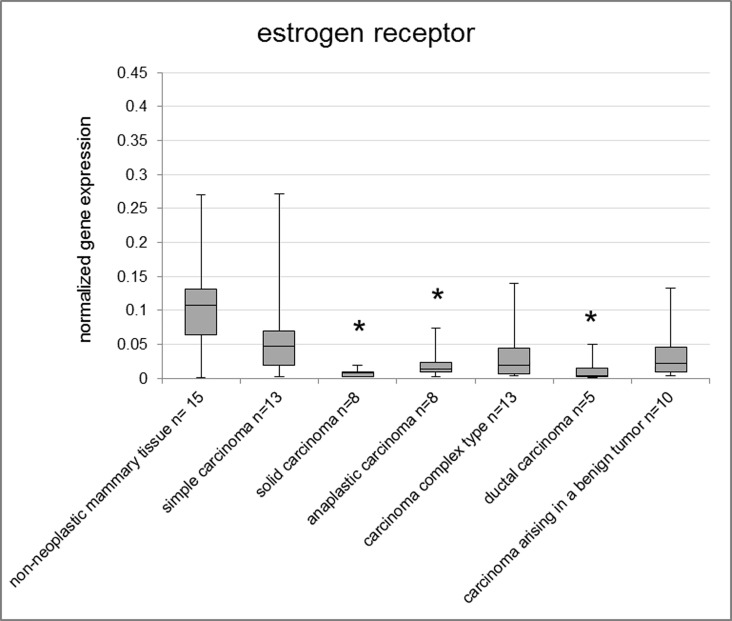
Normalized gene expression of ESR1 in fresh frozen samples. Asterisks indicate statistical significance (* p<0.05; ** p<0.01; *** p<0.001; **** p<0.0001). The box encloses cases within the 25^th^ to the 75^th^ percentiles. The horizontal line within the box represents the median and the upper and lower bars are the largest and lowest observed values. Samples with a value higher than four standard deviations above the mean are not shown in the graph.

The highest ESR1 levels were observed in non-neoplastic mammary tissue and mammary hyperplasia/dysplasia in both fresh frozen and FFPE samples and the lowest in malignant mammary tumors (S1 Fig). Mammary hyperplasia/dysplasia of FFPE samples exhibited significantly higher expression of ESR1 than benign tumors (p = 0.005). Among the histological tumor subtypes, the lowest ESR1 expression of malignant tumors was found in solid carcinomas (fresh frozen p = 0.011; FFPE p = 0.003), ductal carcinomas (fresh frozen p = 0.015; FFPE p = 0.0007), and anaplastic carcinomas (fresh frozen p = 0.014), compared to the non-neoplastic mammary tissue (fresh frozen) and lobular hyperplasias of the mammary gland (FFPE), respectively (Fig 1 and S5 Fig). Moreover, simple tubular carcinomas (p = 0.015), complex carcinomas (p = 0.038), carcinomas and malignant myoepitheliomas (p = 0.01) and intraductal papillary carcinomas (p = 0.006) of FFPE samples exhibited significantly lower ESR1 gene expression than the group of lobular hyperplasia (S5 Fig).

**Fig 2:**
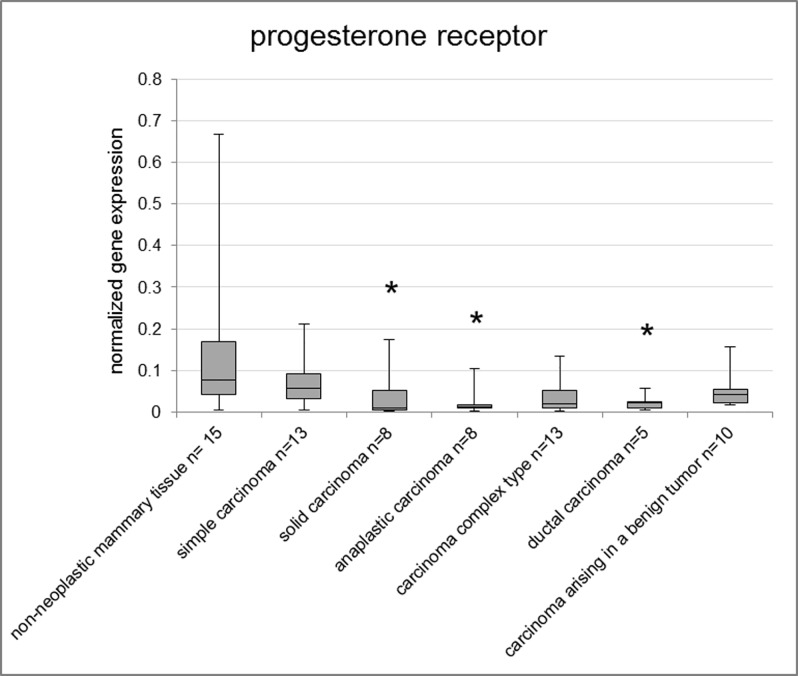
Normalized gene expression of PGR in fresh frozen samples. Asterisks indicate statistical significance (* p<0.05; ** p<0.01; *** p<0.001; **** p<0.0001). The box encloses cases within the 25^th^ to the 75^th^ percentiles. The horizontal line within the box represents the median and the upper and lower bars are the largest and lowest observed values. Samples with a value higher than four standard deviations above the mean are not shown in the graph.

PGR expression levels were significantly lower in solid carcinomas (p = 0.042), anaplastic carcinomas (p = 0.011) and ductal carcinomas (p = 0.036) than the non-neoplastic mammary tissue of fresh frozen samples (Fig 2) when comparing the histological subtypes. The malignant subtypes of FFPE samples showed no significant differences in PGR gene expression compared to the group of lobular hyperplasia (S6 Fig).

**Fig 3:**
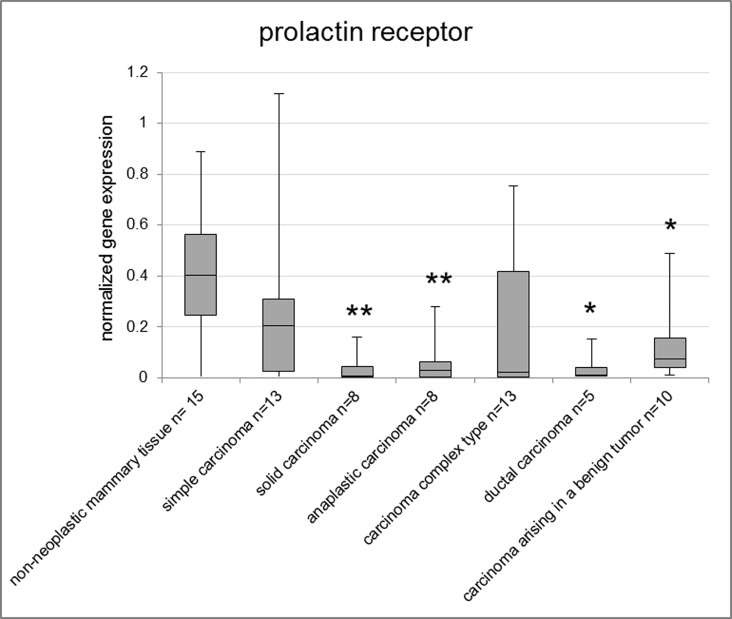
Normalized gene expression of PRLR in fresh frozen samples. Asterisks indicate statistical significance (* p<0.05; ** p<0.01; *** p<0.001; **** p<0.0001). The box encloses cases within the 25^th^ to the 75^th^ percentiles. The horizontal line within the box represents the median and the upper and lower bars are the largest and lowest observed values. Samples with a value higher than four standard deviations above the mean are not shown in the graph.

The highest PRLR levels were observed in non-neoplastic mammary tissue and mammary hyperplasia/dysplasia in both fresh frozen and FFPE samples and the lowest in malignant mammary tumors (S3 Fig). Non-neoplastic tissue of fresh frozen samples exhibited significantly higher expression of PRLR than benign tumors (p = 0.005). Among the histological subtypes, solid carcinoma (p = 0.004), anaplastic carcinoma (p = 0.009), ductal carcinoma (p = 0.018) and carcinoma arising in a benign tumor (p = 0.049) exhibited significantly lower PRLR expression levels than non-neoplastic mammary tissue of fresh frozen samples (Fig 3). All malignant subtypes of the FFPE samples showed significantly lower PRLR gene expression of PRLR than the lobular hyperplasias (S7 Fig). The highest expression levels were found in solid carcinomas (p = 0.002) and ductal carcinomas (p = 0.0009).

**Fig 4:**
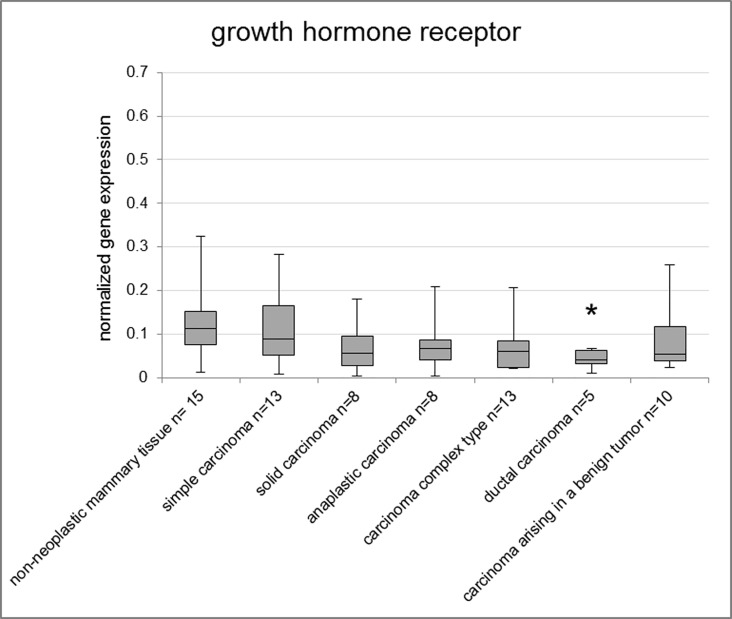
Normalized gene expression of GHR in fresh frozen samples. Asterisks indicate statistical significance (* p<0.05; ** p<0.01; *** p<0.001; **** p<0.0001). The box encloses cases within the 25^th^ to the 75^th^ percentiles. The horizontal line within the box represents the median and the upper and lower bars are the largest and lowest observed values. Samples with a value higher than four standard deviations above the mean are not shown in the graph.

The lowest GHR expression among the histological subtypes of malignant tumors in the fresh frozen samples (Fig 4) was found in ductal carcinomas (p = 0.036). In FFPE samples simple carcinomas (p = 0.044), solid carcinomas (p = 0.005), carcinoma and malignant myoepithelioma (p = 0.036) and ductal carcinomas (p = 0.043) exhibited significantly lower GHR gene expression compared to the group of lobular hyperplasia (S8 Fig).

#### Association between hormone receptor gene expression and invasive growth, castration status and breed

Malignant tumors with invasive growth of FFPE samples showed significantly lower gene expression of hormone receptors than tumors that were not invasive (ESR1 p = 0.014; PGR p = 0.033; PRLR p = 0.0006; GHR p = 0.011, respectively).

No significant differences were found between neutered or intact females and the steroid hormone receptor levels. ESR1 gene expression in malignant mammary tumors was found to be lower in neutered dogs than in intact females in both fresh frozen and FFPE samples. PGR gene expression showed lower levels in malignant mammary tumors of neutered dogs compared to intact females in FFPE samples, but not in fresh frozen samples (Table 3).

**Table 3:** Median of normalized gene expression of ESR1 and PGR in intact (fresh frozen n = 57; FFPE n = 83) and neutered (fresh frozen n = 11; FFPE n = 8) females with malignant mammary tumors.

	Fresh frozen ESR1	FFPE ESR1	Fresh frozen PGR	FFPE PGR
Intact	0.018	0.027	0.029	0.075
Neutered	0.011	0.0103	0.045	0.025

No association was found between the most common breeds (Dachshund, Terrier, Cocker Spaniel, German Shepherd) and hormone receptor levels.

## Discussion

The aim of the present study was to investigate a multiplex bDNA assay for analyzing hormone receptor gene expression in canine mammary tissue comparing fresh frozen and FFPE samples. Until now, most studies evaluating hormone receptors in CMT have used IHC [21–24] referring to the method of choice in human breast cancer. However, results of IHC can be diverse depending on the sample fixation type and time, antibody clones and assay interpretation utilized in different studies [2]. Consequently, the amount of steroid hormone receptor positive tumors varies greatly within studies evaluating CMT [3]. A previous study revealed that, tumors which were initially classified as ESR1 negative by IHC showed differences in ESR1 gene expression [4], thus demonstrating the necessity for a more sensitive method when evaluating hormone receptor expression compared to semi-quantitative IHC. The precise quantitative determination of gene expression using the multiplex bDNA assay detects even low expression of target genes and the sensitivity enables minor varieties to be detected even if the quantity of the sample is limited. Herein, the ESR1, PGR, PRLR and GHR gene expression were analyzed and the results showed all hormone receptors to be significantly lower expressed in malignant mammary tumors compared to the group of reference tissue and benign tumors. These findings are consistent with the literature when measuring ESR1 and PGR on the protein level via IHC [24], as well as gene expression via RT-PCR [25]. Furthermore, the gene expression of ESR1, PGR and PRLR were significantly lower in solid, anaplastic and ductal carcinomas compared to the reference tissue. Solid carcinomas are tumors with a high histological grade (II or III) [26] and anaplastic carcinomas are considered to be the most malignant CMT [20]. Ductal carcinomas showed low ESR1/PGR and PRLR gene expression as well, even though it has been shown that they have a low tendency to invade lymphatic vessel [26]. However, 45% of the ductal carcinomas in the present study showed infiltrative growth, which might explain their low expression levels. Thus, there might be an association between low ESR1, PGR and PRLR expression and the associated prognosis of the tumor. Similarly, a previous study which measured ESR1/PGR in specific histological subtypes in 113 CMT by IHC found an association between low hormone receptor labeling and aggressive neoplastic behavior of the histological subtypes [21]. Similar results have been reported in human breast cancer for both ESR1 and PGR expression [27–28]. For PRLR and GHR no detailed studies on histopathological subtypes or their possible prognostic value in canine mammary tissue have been carried out yet in contrast to human breast cancer. Studies showed an association between PRLR-negative tumors and a higher histological grade [10, 29], whereas results for GHR measured by IHC and RT-PCR suggested no correlation between tumor grade and GHR expression [11] which is comparable to the results in the present study. Castration status should be considered when measuring hormone receptor expression since the production of steroid hormones may have an influence on the formation of receptors [30]. Although ESR1 levels of malignant mammary tumors in the present study were lower in neutered dogs compared to intact females, no statistical significance was found. PR levels were only lower in malignant tumors of the FFPE samples, but not in the fresh frozen specimens. This might be due to small sample numbers (fresh frozen n = 16, FFPE n = 13) considering that in a recent study, ESR1/PGR in malignant mammary tumors was found to be significantly reduced in 30 neutered females compared to 49 intact females with malignant mammary tumors [21]. Thus, the influence of castration time on mammary tumor development and hormone receptor expression needs to be further evaluated. As incidence of CMT might be breed dependent [31], we further hypothesized if there is an association between hormone receptor gene expression and overrepresented breeds. However, no differences could be found when comparing the hormone receptor expression of the overrepresented breeds.

In the present study, FFPE samples were used to enlarge the number of samples, but also to evaluate if they are as reliable as fresh frozen samples for future studies using the multiplex bDNA assay. FFPE samples are widely available from tumor resection and consequent histopathological analysis [32], and therefore present a potential alternative source to fresh frozen samples for retrospective studies. Target-RNA was detected in both fresh frozen and FFPE samples in this study. Only 15 FFPE samples (9% of all FFPE samples measured) had to be excluded due to values below the limit of detection. Interestingly, these samples also showed the lowest yields when isolating the RNA. When grouping the tumors according to their special histological subtype, only the gene expression of PGR was found to vary significantly between fresh frozen and FFPE samples in two of the subtypes (simple tubular carcinoma and carcinoma arising in a benign tumor). As it was not possible to measure the equivalent sample of fresh frozen and FFPE, a variance in the measured sample existed, which might explain the significant differences between the gene expression among the histological subtypes. Nevertheless, the measurement of hormone receptors in the present study with the multiplex bDNA assay was reliable for both fresh frozen and FFPE samples of canine mammary tissue. Therefore, FFPE samples could be a potential source for enlarging the number of samples for future studies. Still, fresh frozen samples proved to be more sensitive with 100% of the samples being analyzable suggesting them to be the sample of choice for individual studies.

In summary, the observations in the present study attest that measuring of hormone receptors with the multiplex bDNA assay is a practicable method for gaining quantitative and detailed information about gene expression in canine mammary tissue for future studies. Nevertheless, IHC still presents a clinically validated method for analyzing the steroid hormone receptor status in human breast cancer [1]. The procedure preserves the morphology of the tissue [2] and therefore allows a direct identification of hormone receptors in the respective tissue in contrast to gene expression studies. Consequently, novel approaches for quantitative gene expression of steroid hormone receptors need to be compared to the standard method. In human breast cancer a high degree of concordance between methods for gene expression analysis and IHC has already been demonstrated [33–34]. Furthermore, cut-off values for receptor negative vs. positive tumors need to be established and have already been determined for ESR1 and PGR in human breast cancer using a bDNA assay [34]. For canine mammary tissue the multiplex bDNA assay still needs to be validated in future studies to elucidate the concordance rate with IHC and to develop cut-off values for receptor negative or positive tissue.

## Conclusion

In conclusion, the multiplex bDNA assay could be a practicable method for future multigene studies in canine mammary tissue. Its advantages include a sensitive quantitative determination for detecting minor varieties and analyzing even small-sized specimens, as well as detecting several targets in one sample [6]. Still, comparison with IHC or quantitative real-time PCR is needed to further validate the presented method. The present study revealed the multiplex bDNA assay to be reliable for both fresh frozen and FFPE samples, but fresh frozen samples proved to be more sensitive especially for individual studies. The evaluated hormone receptors were found to be lower expressed in malignant mammary tumors. Especially for the receptors of estrogen, progesterone and prolactin the results indicate that the expression is depending on the histological subtype and that they might act as possible prognostic markers.

## Supporting Information

S1 Fig Normalized gene expression of ESR1 in the diagnosis groups of fresh frozen and FFPE samples. Asterisks indicate statistical significance (* p<0.05; ** p<0.01; *** p<0.001; **** p<0.0001). The box encloses cases within the 25^th^ to the 75^th^ percentiles. The horizontal line within the box represents the median and the upper and lower bars are the largest and lowest observed values. Samples with a value higher than four standard deviations above the mean are not shown in the graph.
(TIF)

S2 Fig Normalized gene expression of PGR in the diagnosis groups of fresh frozen and FFPE samples. Asterisks indicate statistical significance (* p<0.05; ** p<0.01; *** p<0.001; **** p<0.0001). The box encloses cases within the 25^th^ to the 75^th^ percentiles. The horizontal line within the box represents the median and the upper and lower bars are the largest and lowest observed values. Samples with a value higher than four standard deviations above the mean are not shown in the graph.
(TIF)

S3 Fig Normalized gene expression of PRLR in the diagnosis groups of fresh frozen and FFPE samples. Asterisks indicate statistical significance (* p<0.05; ** p<0.01; *** p<0.001; **** p<0.0001. The box encloses cases within the 25^th^ to the 75^th^ percentiles. The horizontal line within the box represents the median and the upper and lower bars are the largest and lowest observed values. Samples with a value higher than four standard deviations above the mean are not shown in the graph.
(TIF)

S4 Fig Normalized gene expression of GHR in the diagnosis groups of fresh frozen and FFPE samples. Asterisks indicate statistical significance (* p<0.05; ** p<0.01; *** p<0.001; **** p<0.0001. The box encloses cases within the 25^th^ to the 75^th^ percentiles. The horizontal line within the box represents the median and the upper and lower bars are the largest and lowest observed values. Samples with a value higher than four standard deviations above the mean are not shown in the graph.
(TIF)

S5 Fig Normalized gene expression of ESR1 in histopathological subtypes of FFPE samples. Asterisks indicate statistical significance (* p<0.05; ** p<0.01; *** p<0.001; **** p<0.0001) in comparison to the non-neoplastic tissue. The box encloses cases within the 25^th^ to the 75^th^ percentiles. The horizontal line within the box represents the median and the upper and lower bars are the largest and lowest observed values. Samples with a value higher than four standard deviations above the mean are not shown in the graph.
(TIF)

S6 Fig Normalized gene expression of PGR in histopathological subtypes of FFPE samples. Asterisks indicate statistical significance (* p<0.05; ** p<0.01; *** p<0.001; **** p<0.0001) in comparison to the non-neoplastic tissue. The box encloses cases within the 25^th^ to the 75^th^ percentiles. The horizontal line within the box represents the median and the upper and lower bars are the largest and lowest observed values. Samples with a value higher than four standard deviations above the mean are not shown in the graph.
(TIF)

S7 Fig Normalized gene expression of PRLR in histopathological subtypes of FFPE samples. Asterisks indicate statistical significance (* p<0.05; ** p<0.01; *** p<0.001; **** p<0.0001) in comparison to the non-neoplastic tissue. The box encloses cases within the 25^th^ to the 75^th^ percentiles. The horizontal line within the box represents the median and the upper and lower bars are the largest and lowest observed values. Samples with a value higher than four standard deviations above the mean are not shown in the graph.
(TIF)

S8 Fig Normalized gene expression of GHR in histopathological subtypes of FFPE samples. Asterisks indicate statistical significance (* p<0.05; ** p<0.01; *** p<0.001; **** p<0.0001) in comparison to the non-neoplastic tissue. The box encloses cases within the 25^th^ to the 75^th^ percentiles. The horizontal line within the box represents the median and the upper and lower bars are the largest and lowest observed values. Samples with a value higher than four standard deviations above the mean are not shown in the graph.
(TIF)

S1 Table Median and range of normalized hormone receptor gene expression in fresh frozen samples. (TIF)

S2 Table Median and range of normalized hormone receptor gene expression in FFPE samples. (TIF)

S3 Table Raw data of the Luminex and normalized gene expression of fresh frozen and FFPE samples. (XLSX)
