# Supplementary figures and images for: Hormone Receptor Expression Analyses in Neoplastic and Non-Neoplastic Canine Mammary Tissue by a Bead Based Multiplex Branched DNA Assay: A Gene Expression Study in Fresh Frozen and Formalin-Fixed, Paraffin-Embedded Samples
Source: PLoS One. 2016 Sep 20;11(9):e0163311. doi: 10.1371/journal.pone.0163311 (PMC5029807; doi:10.1371/journal.pone.0163311)

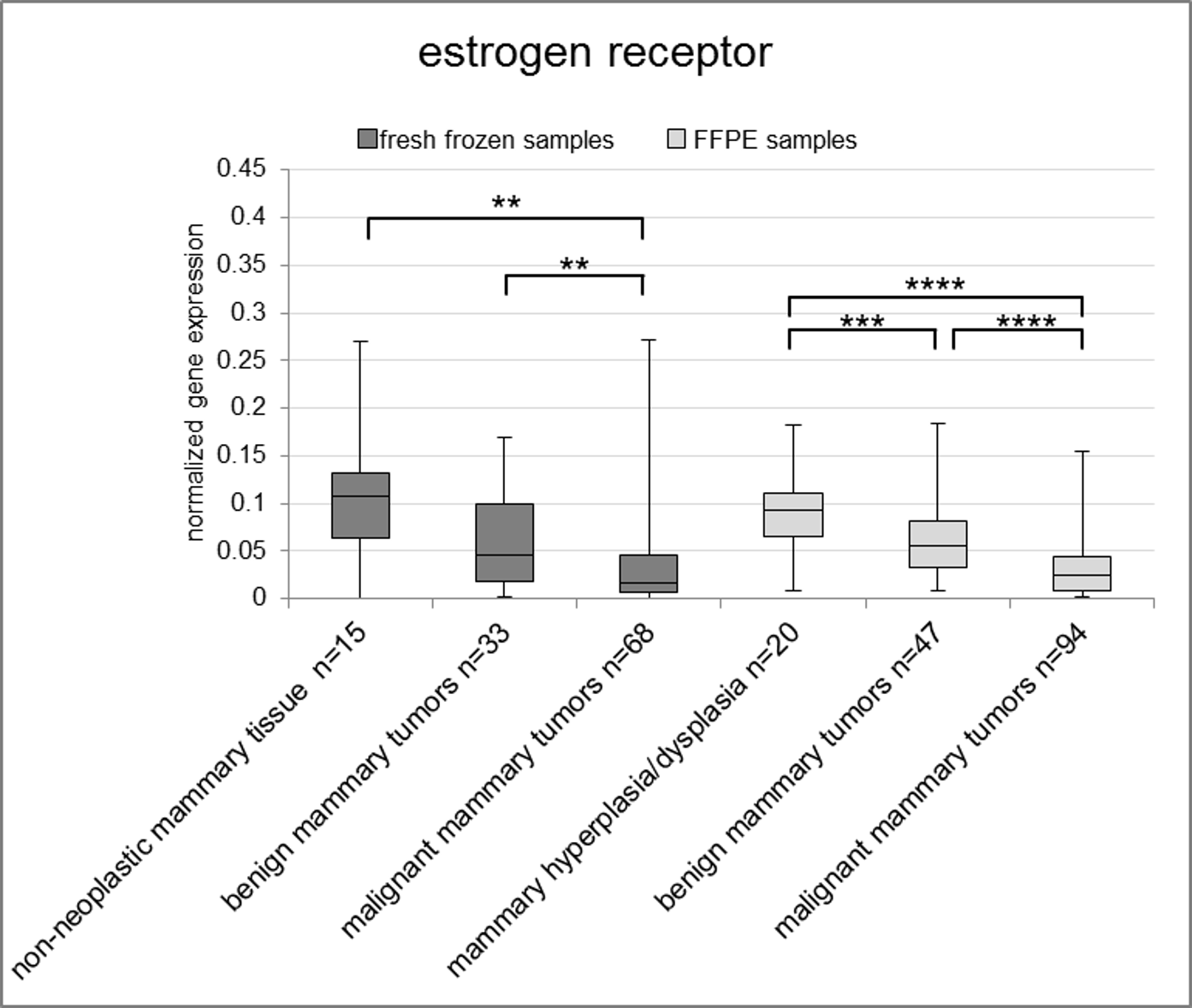

Supplement: S1 Fig — Asterisks indicate statistical significance (* p<0.05; ** p<0.01; *** p<0.001; **** p<0.0001). The box encloses cases within the 25th to the 75th percentiles. The horizontal line within the box represents the median and the upper and lower bars are the largest and lowest observed values. Samples with a value higher than four standard deviations above the mean are not shown in the graph. (TIF) [file pone.0163311.s001.tif]

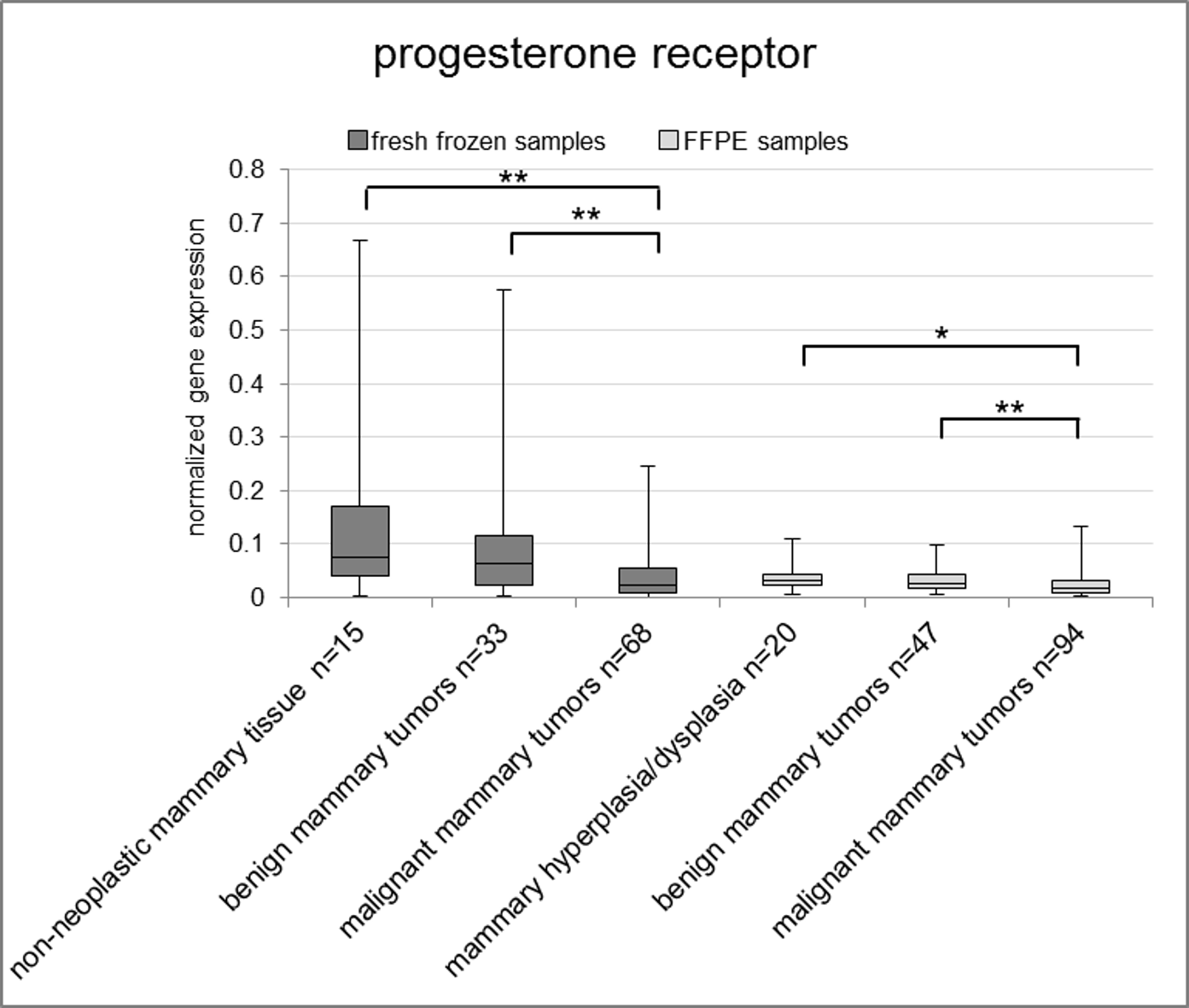

Supplement: S2 Fig — Asterisks indicate statistical significance (* p<0.05; ** p<0.01; *** p<0.001; **** p<0.0001). The box encloses cases within the 25th to the 75th percentiles. The horizontal line within the box represents the median and the upper and lower bars are the largest and lowest observed values. Samples with a value higher than four standard deviations above the mean are not shown in the graph. (TIF) [file pone.0163311.s002.tif]

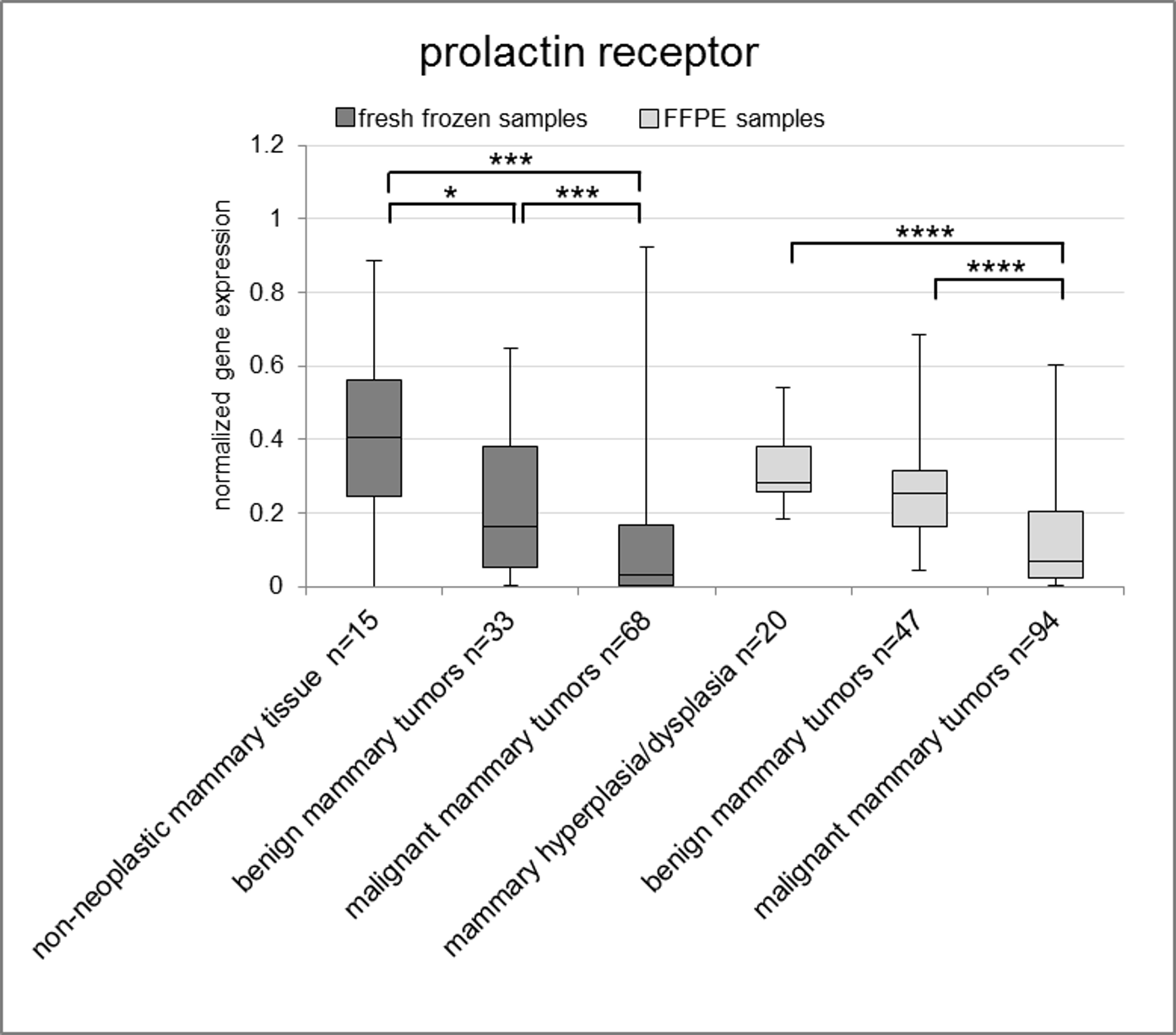

Supplement: S3 Fig — Asterisks indicate statistical significance (* p<0.05; ** p<0.01; *** p<0.001; **** p<0.0001. The box encloses cases within the 25th to the 75th percentiles. The horizontal line within the box represents the median and the upper and lower bars are the largest and lowest observed values. Samples with a value higher than four standard deviations above the mean are not shown in the graph. (TIF) [file pone.0163311.s003.tif]

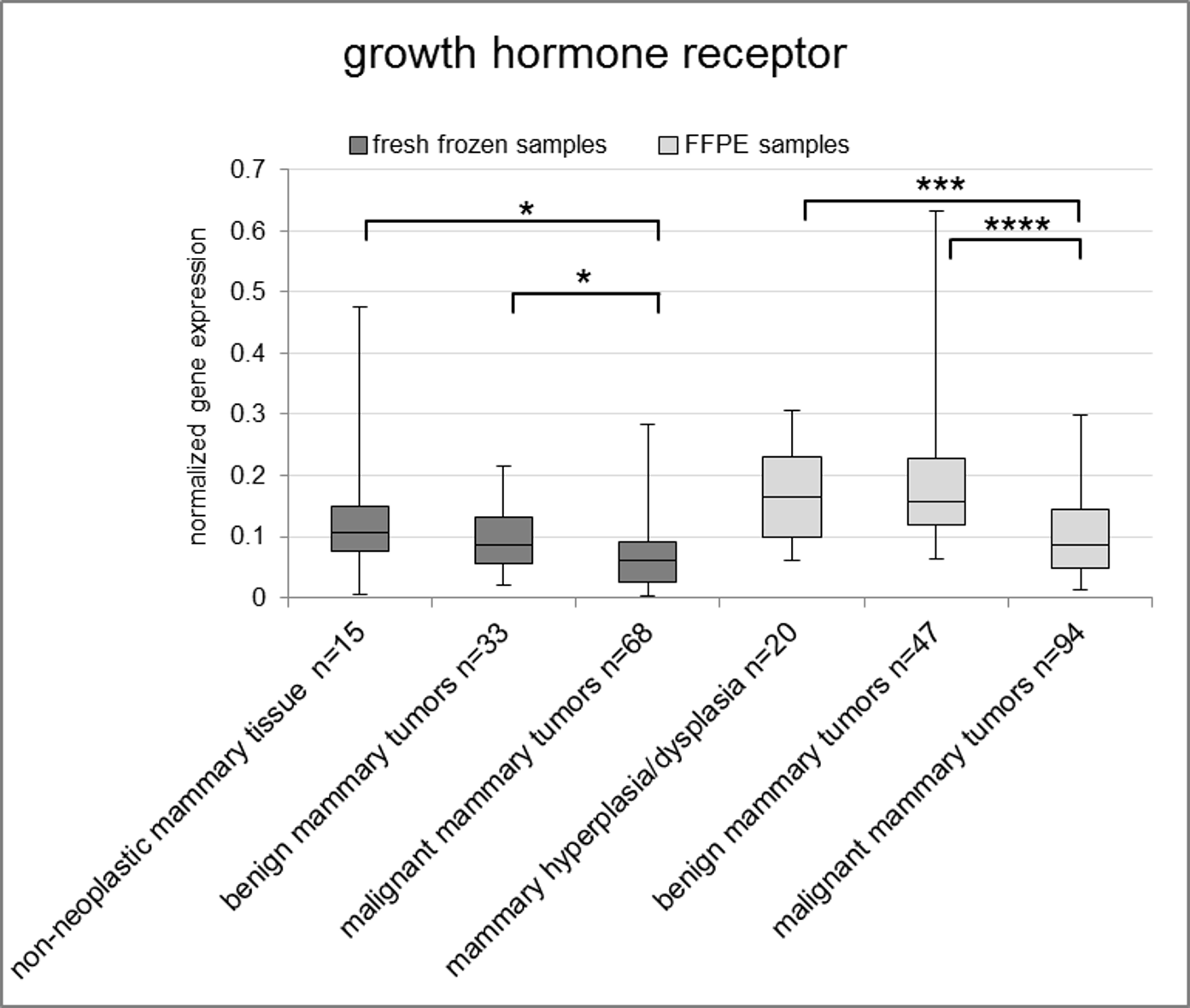

Supplement: S4 Fig — Asterisks indicate statistical significance (* p<0.05; ** p<0.01; *** p<0.001; **** p<0.0001. The box encloses cases within the 25th to the 75th percentiles. The horizontal line within the box represents the median and the upper and lower bars are the largest and lowest observed values. Samples with a value higher than four standard deviations above the mean are not shown in the graph. (TIF) [file pone.0163311.s004.tif]

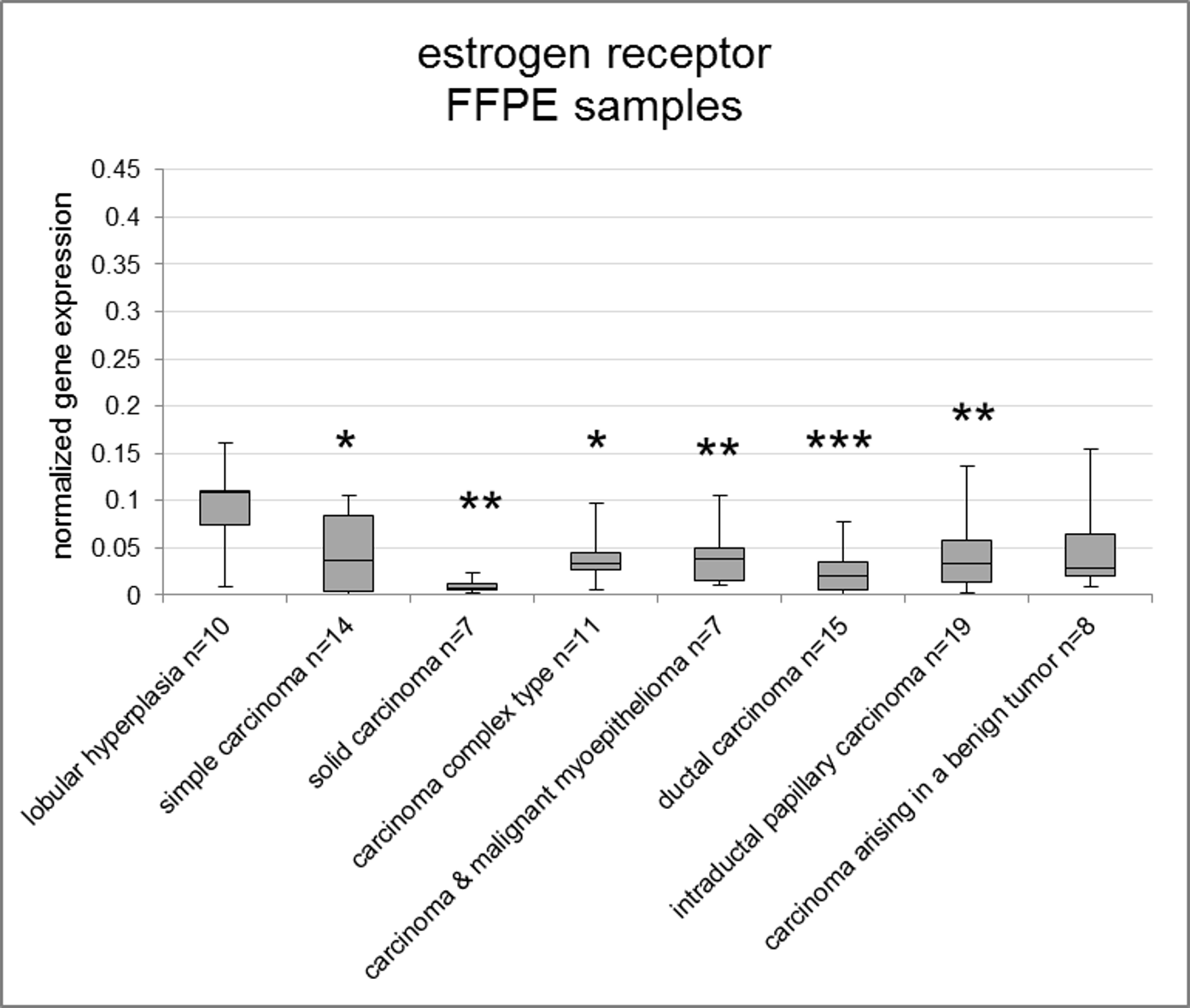

Supplement: S5 Fig — Asterisks indicate statistical significance (* p<0.05; ** p<0.01; *** p<0.001; **** p<0.0001) in comparison to the non-neoplastic tissue. The box encloses cases within the 25th to the 75th percentiles. The horizontal line within the box represents the median and the upper and lower bars are the largest and lowest observed values. Samples with a value higher than four standard deviations above the mean are not shown in the graph. (TIF) [file pone.0163311.s005.tif]

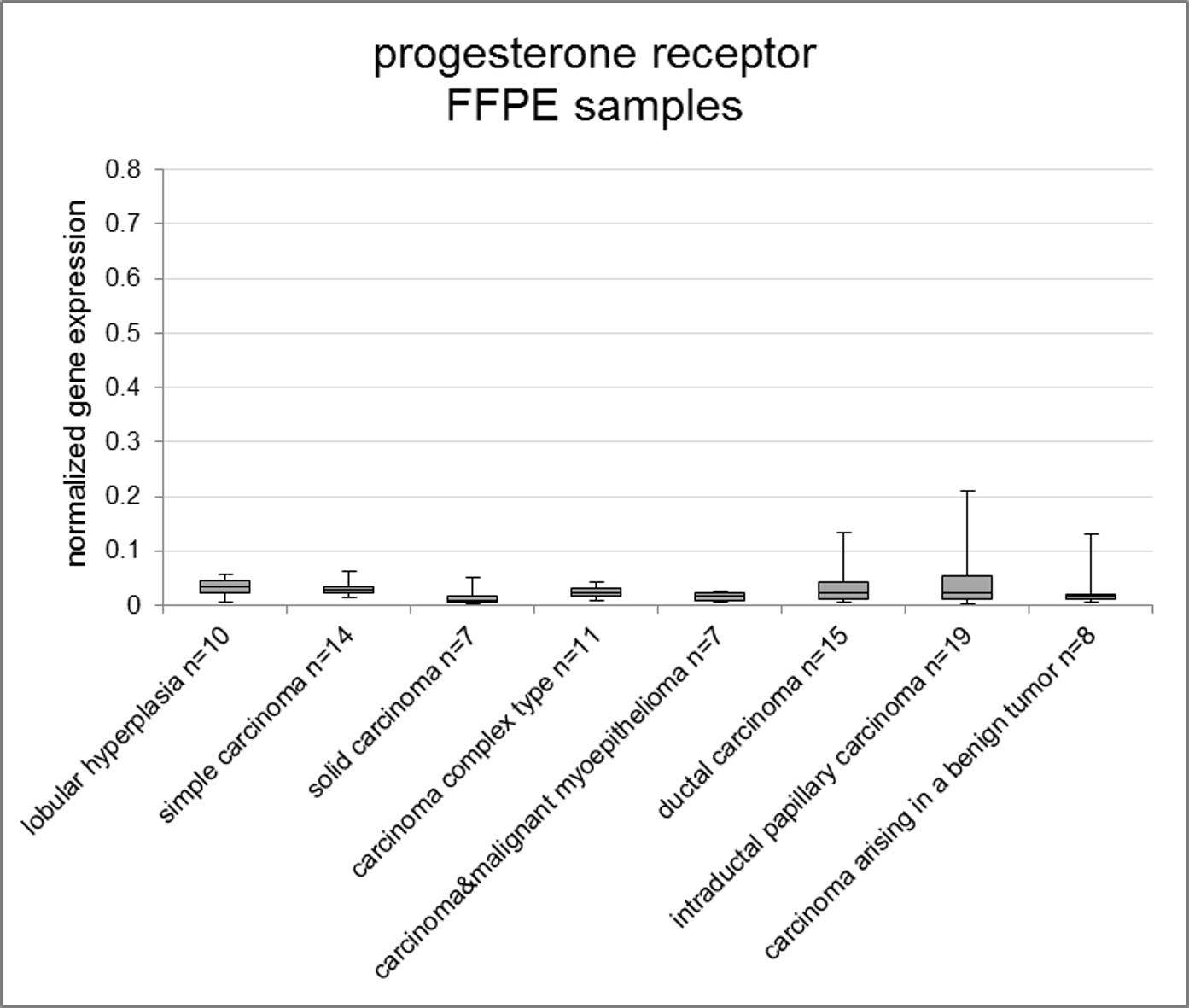

Supplement: S6 Fig — Asterisks indicate statistical significance (* p<0.05; ** p<0.01; *** p<0.001; **** p<0.0001) in comparison to the non-neoplastic tissue. The box encloses cases within the 25th to the 75th percentiles. The horizontal line within the box represents the median and the upper and lower bars are the largest and lowest observed values. Samples with a value higher than four standard deviations above the mean are not shown in the graph. (TIF) [file pone.0163311.s006.tif]

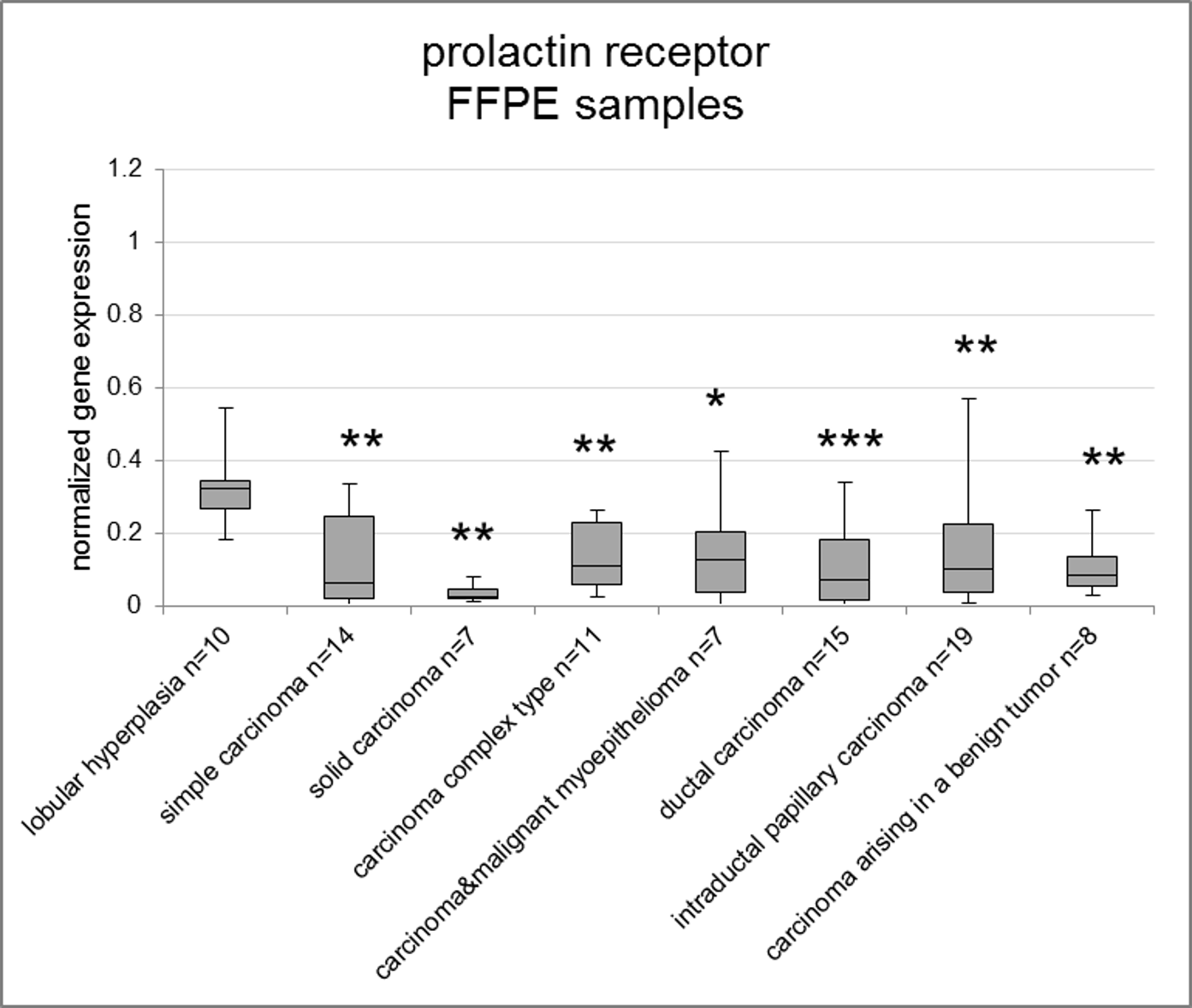

Supplement: S7 Fig — Asterisks indicate statistical significance (* p<0.05; ** p<0.01; *** p<0.001; **** p<0.0001) in comparison to the non-neoplastic tissue. The box encloses cases within the 25th to the 75th percentiles. The horizontal line within the box represents the median and the upper and lower bars are the largest and lowest observed values. Samples with a value higher than four standard deviations above the mean are not shown in the graph. (TIF) [file pone.0163311.s007.tif]

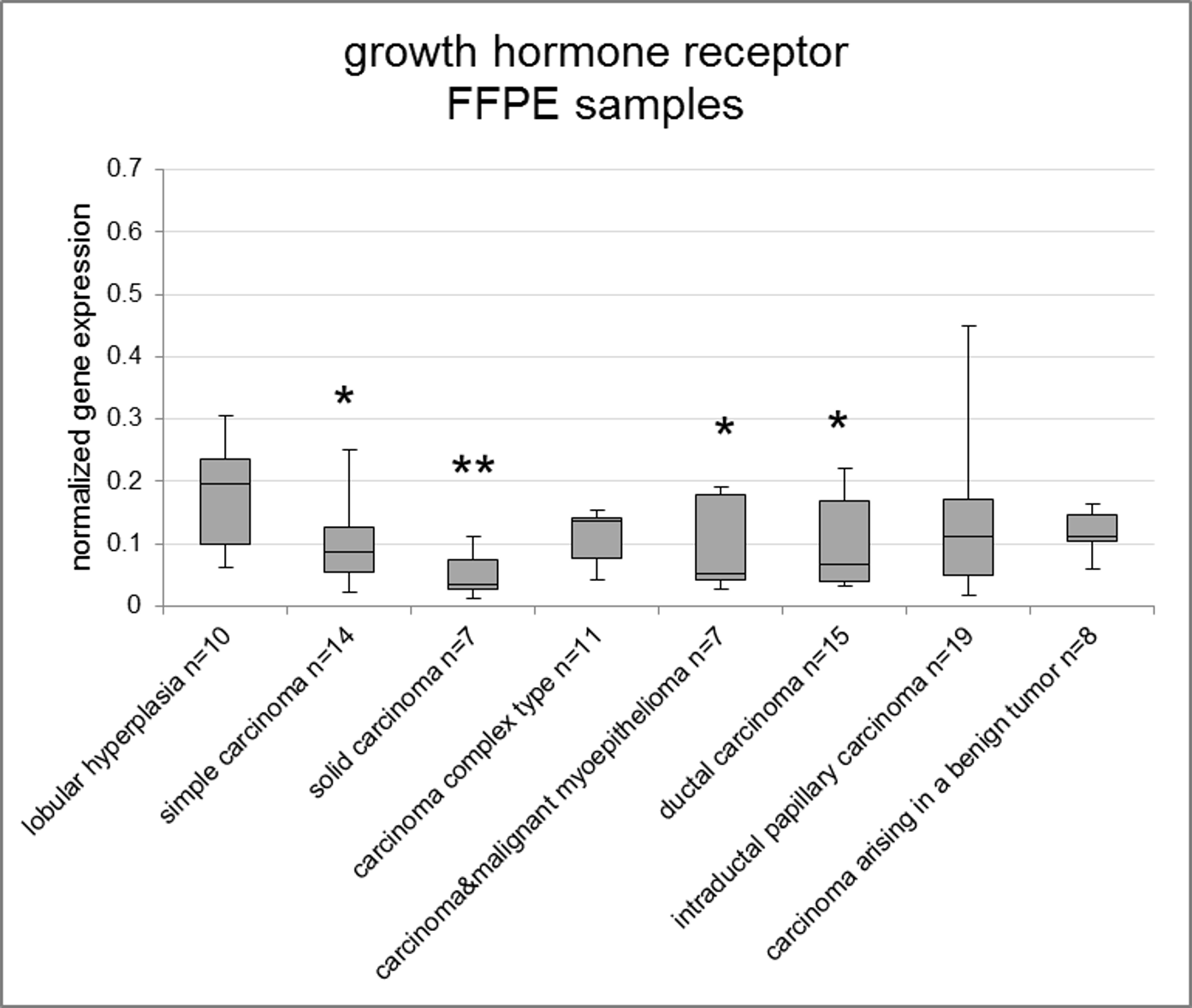

Supplement: S8 Fig — Asterisks indicate statistical significance (* p<0.05; ** p<0.01; *** p<0.001; **** p<0.0001) in comparison to the non-neoplastic tissue. The box encloses cases within the 25th to the 75th percentiles. The horizontal line within the box represents the median and the upper and lower bars are the largest and lowest observed values. Samples with a value higher than four standard deviations above the mean are not shown in the graph. (TIF) [file pone.0163311.s008.tif]

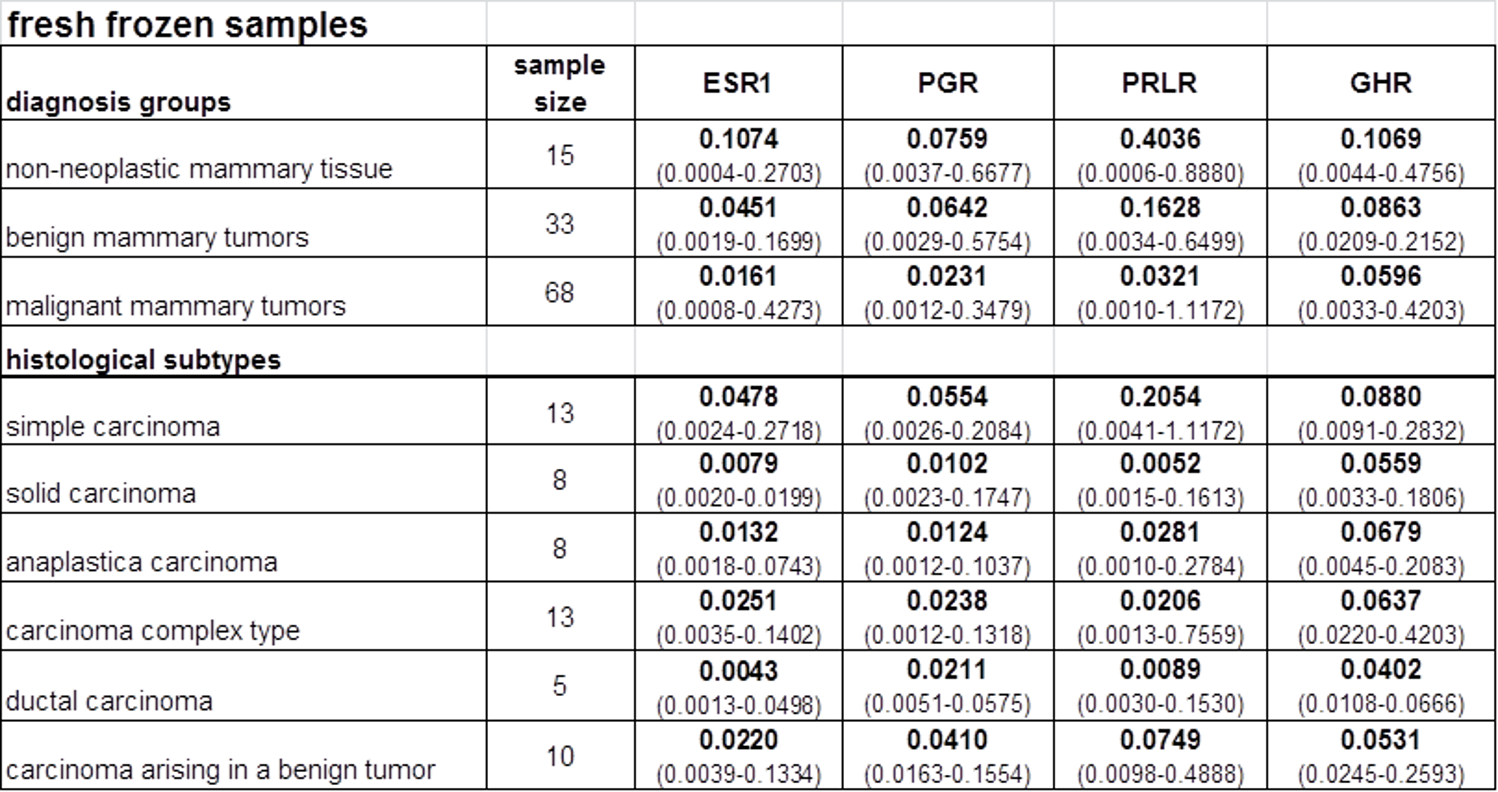

Supplement: S1 Table — (TIF) [file pone.0163311.s009.tif]

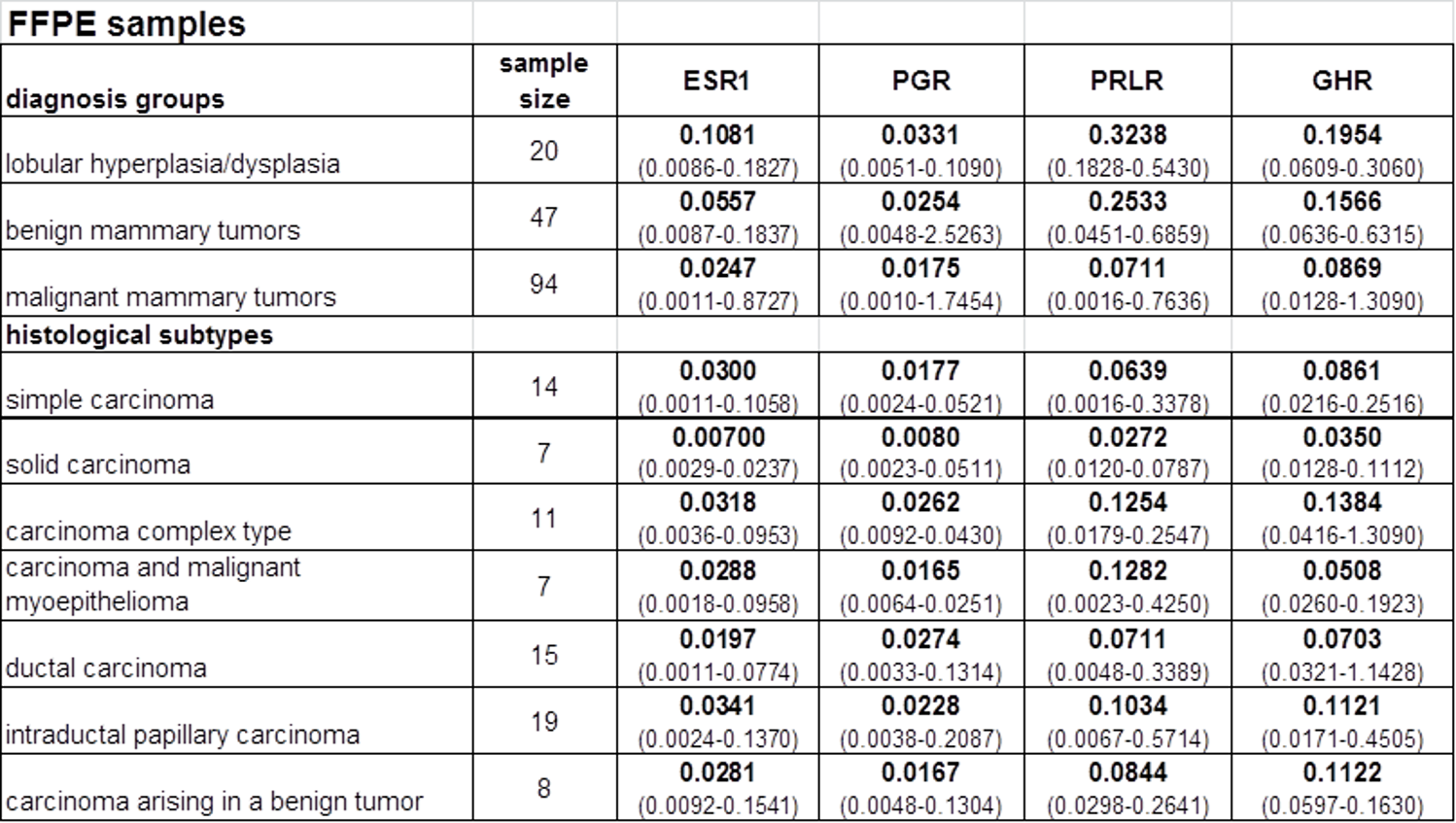

Supplement: S2 Table — (TIF) [file pone.0163311.s010.tif]
